# Supplementary material for: Chikungunya Virus Asian Lineage Infection in the Amazon Region Is Maintained by Asiatic and Caribbean-Introduced Variants
Source: Viruses. 2022 Jun 30;14(7):1445. doi: 10.3390/v14071445 (PMC9319912; doi:10.3390/v14071445)
Supplement: Supplementary file 1 [file viruses-14-01445-s001.zip › viruses-1744273-supplementary.pdf]

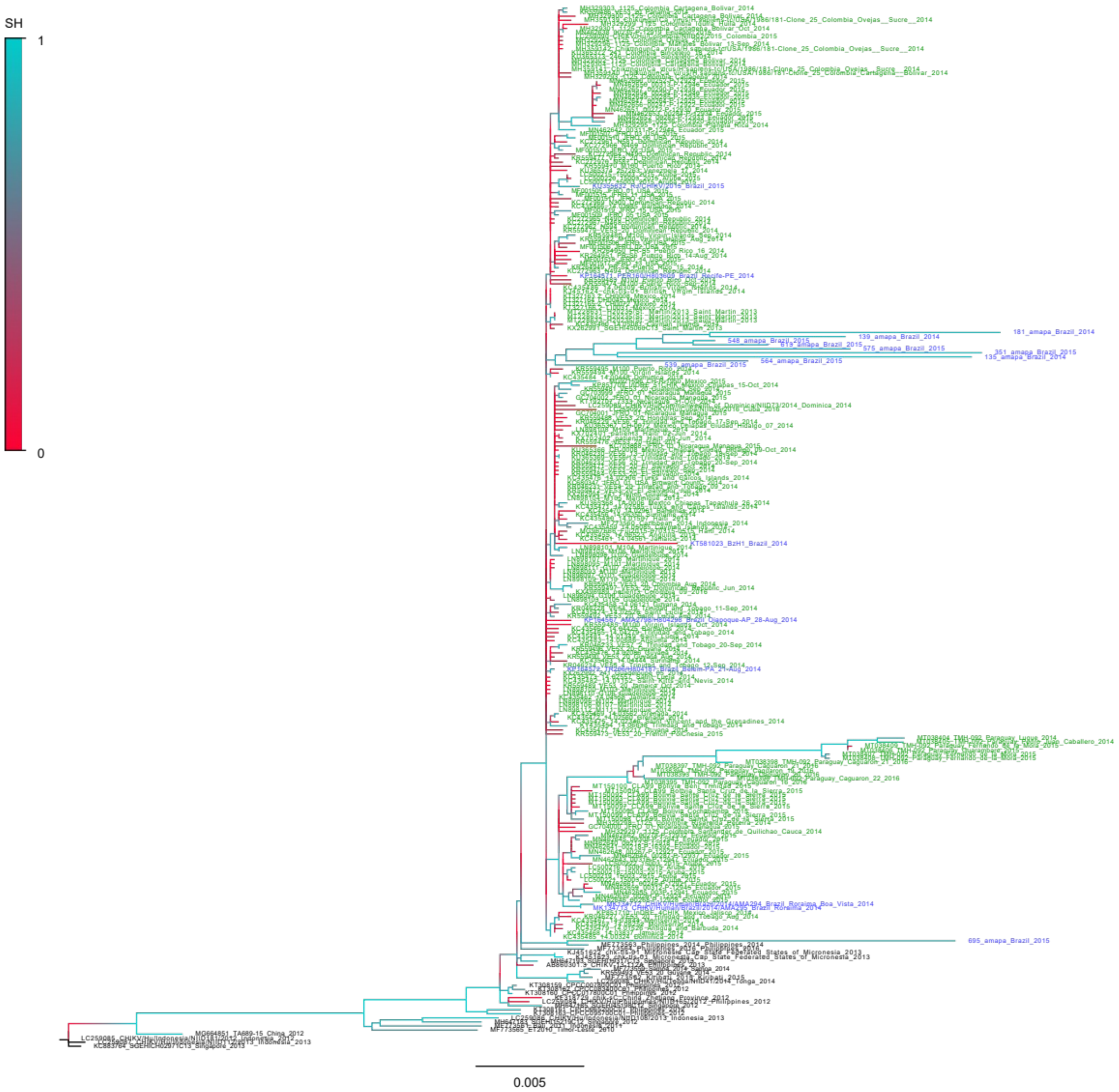
SH
1
0
0.005
181_amapa_Brazil_2014
139_amapa_Brazil_2014
575_amapa_Brazil_2015
351_amapa_Brazil_2015
135_amapa_Brazil_2014
584_amapa_Brazil_2015
539_amapa_Brazil_2015
KT581023_BzH1_Brazil_2014
695_amapa_Brazil_2015

Figure S1. Maximum likelihood tree of CHIKV-Asian lineage. The unrooted tree was constructed using reference genomes of CHIKV-Asian lineage. Sequences from Brazil are indicated in blue. Sequences from Caribbean countries are in green color and sequences from South Asia/Oceania are in black color. The cluster composed by Caribbean sequences plus almost all Brazilian sequences is labeled. The Brazilian sequence related with a Philippine sequences is highlighted. The branch support is indicated by a color scale of 0 to 1, and is based on the Shimodaira-Hasegawa-like test. The tree was inferred using the TN-93 model plus gamma correction. Horizontal bar indicate the nucleotide substitution per base.

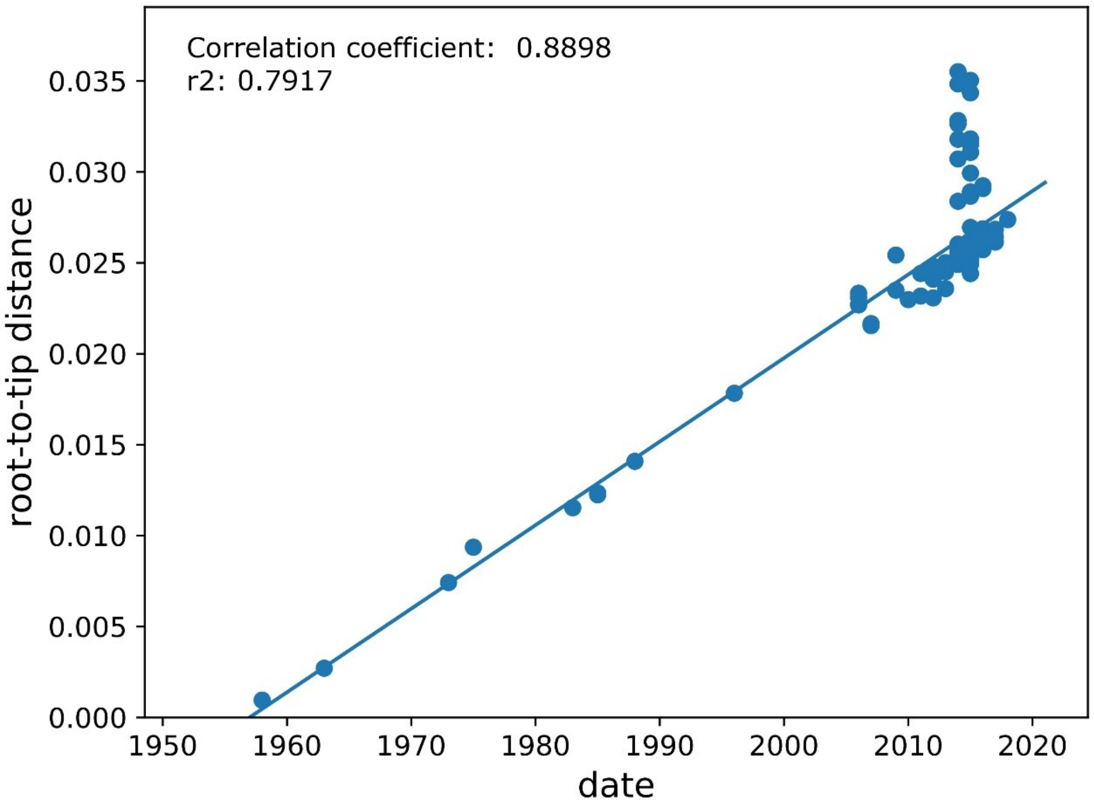

Figure S2. Linear regression analysis. root-to-tip genetic distance against year of sampling fof 297 CHIKV Asian lineage sequences. Linear regression was performed and the trend line and R-squared value (r2) are indicated.

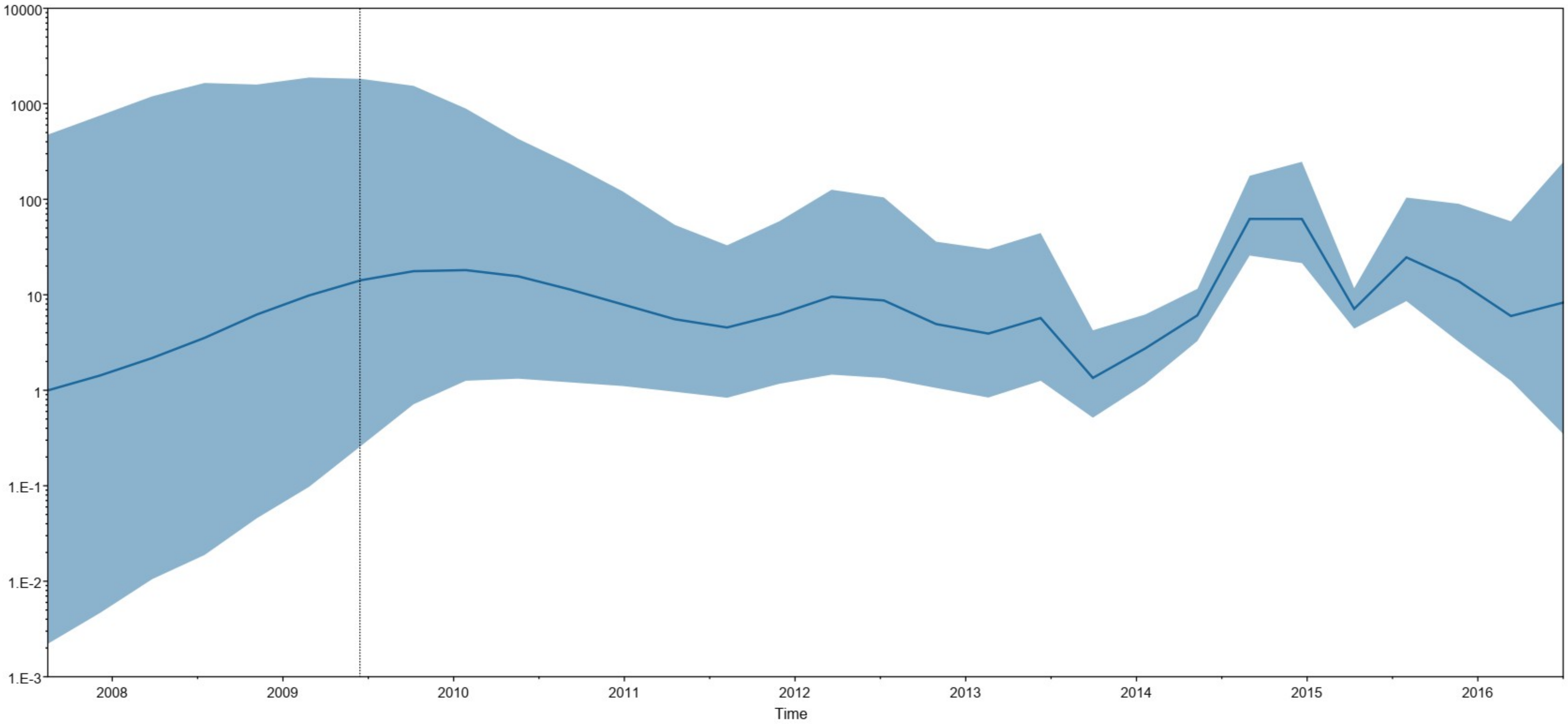

Figure S3. Skygrid demographic of CHIKV. The reconstruction plot, based on relaxed-clock coalescent analysis. X-axis represents time in years, while Y-axis shows the Effective population size at time t (Ne(t)). The thick black line represents the median, while the blue band represents 95% highest posterior density (HPD) intervals.
